# ATF6 is required for efficient rhodopsin clearance and retinal homeostasis in the P23H rho retinitis pigmentosa mouse model

**DOI:** 10.1038/s41598-021-95895-7

**Published:** 2021-08-11

**Authors:** Eun-Jin Lee, Priscilla Chan, Leon Chea, Kyle Kim, Randal J. Kaufman, Jonathan H. Lin

**Affiliations:** 1 Department of Ophthalmology, Stanford University, Palo Alto, CA USA; 2 Department of Pathology, Stanford University, Palo Alto, CA USA; 3 VA Palo Alto Healthcare System, Palo Alto, CA USA; 4 USC ROSKI Eye Institute and Department of Ophthalmology, Keck School of Medicine, University of Southern California, Los Angeles, CA USA; 5 Department of Neurology, Keck School of Medicine, University of Southern California, Los Angeles, CA USA; 6 Degenerative Diseases Program, Sanford-Burnham-Prebys Medical Discovery Institute, La Jolla, CA USA; 7 School of Medicine, Stanford University, 300 Pasteur Dr. L235, Palo Alto, CA 94305 USA

**Keywords:** Biochemistry, Molecular medicine, Pathogenesis

## Abstract

Retinitis Pigmentosa (RP) is a blinding disease that arises from loss of rods and subsequently cones. The P23H rhodopsin knock-in (P23H-KI) mouse develops retinal degeneration that mirrors RP phenotype in patients carrying the orthologous variant. Previously, we found that the P23H rhodopsin protein was degraded in P23H-KI retinas, and the Unfolded Protein Response (UPR) promoted P23H rhodopsin degradation in heterologous cells in vitro. Here, we investigated the role of a UPR regulator gene, activating transcription factor 6 (*Atf6*), in rhodopsin protein homeostasis in heterozygous P23H rhodopsin (*Rho*^+*/P23H*^) mice. Significantly increased rhodopsin protein levels were found in *Atf6*^*−/−*^*Rho*^+*/P23H*^ retinas compared to *Atf6*^+*/−*^*Rho*^+*/P23H*^ retinas at early ages (~ P12), while *rhodopsin* mRNA levels were not different. The IRE1 pathway of the UPR was hyper-activated in young *Atf6*^*−/−*^*Rho*^+*/P23H*^ retinas, and photoreceptor layer thickness was unchanged at this early age in *Rho*^+*/P23H*^ mice lacking *Atf6*. By contrast, older *Atf6*^*−/−*^*Rho*^+*/P23H*^ mice developed significantly increased retinal degeneration in comparison to *Atf6*^+*/−*^*Rho*^+*/P23H*^ mice in all retinal layers, accompanied by reduced rhodopsin protein levels. Our findings demonstrate that *Atf6* is required for efficient clearance of rhodopsin protein in rod photoreceptors expressing P23H rhodopsin, and that loss of *Atf6* ultimately accelerates retinal degeneration in P23H-KI mice.

## Introduction

Retinitis pigmentosa (RP) is a group of retinal degenerative diseases that leads to irreversible blindness with a worldwide prevalence of 1:4000^1,2^. RP causes progressive retinal degeneration that first results in the loss of rod photoreceptors which then results in the loss of cone photoreceptors, and gives rise to initial night blindness and loss of peripheral vision. Hundreds of gene mutations have been found to cause heritable forms of RP (RetNet, Retinal Information Network, at https://sph.uth.edu/retnet/disease.htm). Mutations in the rhodopsin gene are a common cause of autosomal dominant RP (adRP). More than 150 distinct rhodopsin mutations have been identified in adRP (RetNet[Retinal Information Network- www.sph.uth.tmc.edu/Retnet]^2–4^. Most mutations introduce missense changes throughout the rhodopsin protein coding region; the most common mutation in the USA is the substitution of proline to histidine at codon 23 (P23H) in the extracellular N-terminal domain, accounting for 15–20% of all adRP cases in the USA^5–7^.

The endoplasmic reticulum (ER) is vital for membrane protein packaging, secretion, and folding, and, consequently, protein misfolding in the ER can disrupt ER function and lead to “ER stress”. In response to ER stress, cells activate the Unfolded Protein Response (UPR), a cellular homeostatic mechanism that reduces ER stress by promoting the degradation of misfolded proteins and slowing new protein synthesis^8^. The UPR is controlled by 3 ER resident transmembrane proteins, inositol-requiring enzyme 1 (IRE1), activating transcription factor 6 (ATF6), and protein kinase RNA-like endoplasmic reticulum kinase (PERK). In response to ER stress, IRE1 activates its kinase and RNase functions, initiating the nonconventional splicing of X-box binding protein 1 (*XBP-1*) mRNA^9–11^. Spliced *XBP-1* (*XBP-1s*) mRNA encodes a transcription activator that induces expression of ER chaperones and ER-associated degradation (ERAD) components that remove and degrade misfolded proteins via the ubiquitin–proteasome system^12,13^. The PERK arm of the UPR regulates protein synthesis. Upon ER stress, PERK oligomerizes via its cytoplasmic domain, which leads to phosphorylation of eukaryotic initiation factor 2α (eIF2α) and, as a result, attenuation of protein translation^8,14–16^. Under prolonged ER stress, PERK promotes cell death by activating stress-responsive transcription factors such as activating transcription factor 4 (ATF4) and CCAAT/enhancer-binding protein homologous protein (CHOP)^17,18^. ATF6 undergoes proteolytic processing in response to ER stress, releasing its cytosolic bZIP-containing transcription factor domain (ATF6f), which then travels to the nucleus to upregulate genes involved in ER protein folding and ERAD (that significantly overlap with the transcriptional targets of XBP-1s)^19–21^. Thus, activation of the UPR ultimately promotes cell viability and homeostasis by reducing misfolded protein levels.

Here, we used the P23H-KI mouse model to evaluate the UPR’s function in rod photoreceptors in the eye and in the pathogenesis/progression of retinal degeneration arising from misfolded rhodopsin proteins^22,23^. Within two weeks of age, photoreceptor and retinal degeneration begin in the *Rho*^+*/P23H*^ mice^22,23^. At post-natal day 30 (P30) and P60, degeneration continues with outer nuclear layer (ONL) thinning, loss of rhodopsin protein levels, and poorer ERG responses by a-wave and b-wave analysis^22–24^. In addition, *Rho*^+*/P23H*^ mice show progressive shortening of the rod outer segment and rod inner segment at P30 and P60^22–24^. In healthy retinas, rhodopsin is translated at the ER membrane and, when correctly folded, rhodopsin is exported to the outer segment of rod photoreceptor cells to initiate phototransduction in response to light. In contrast, biochemical and cellular studies reveal that P23H rhodopsin is misfolded, retained in the ER, and aggregates; as a result, P23H rhodopsin may be a source of ER stress and cause activation of the UPR^6,25^. The role of P23H rhodopsin as an ER stress inducer in rod photoreceptors is supported by the activation of the IRE1 pathway in the *Rho*^+*/P23H*^ mice^26^, which was found using an IRE1-reporter *ER stress activated indicator* (*ERAI*) transgenic mouse crossed with P23H-KI mice. The *Rho*^+*/P23H*^ mice also showed significant increase of *XBP-1s* mRNA and XBP-1s targets, such as DNA damage inducible transcript 3 (*Ddit3), SEC24 homolog d (Sec24d),* and homocysteine inducible ER protein with ubiquitin like domain 1 (*Herpud1)*, *in Rho*^+*/P23H*^ mice at P30, P60, P90, and P120^26^. These findings demonstrate increased IRE1 activity in rods expressing the P23H misfolded rhodopsin. We therefore proposed that misfolded rhodopsin generated by the P23H mutation activates the UPR, and that in turn may alleviate ER stress caused by P23H rhodopsin through protein clearance mechanisms such as ERAD.

Previously, we demonstrated that chemical-genetic activation of either IRE1 (to produce XBP-1s) or ATF6f expression promoted P23H rhodopsin protein degradation when expressed in heterologous HEK293 cells, while sparing wild-type rhodopsin protein^22,27^. By contrast, PERK signaling negatively impacted both misfolded and wild-type rhodopsin protein levels^27^. These findings suggested that IRE1, XBP-1s, and ATF6 are important for removing P23H rhodopsin from photoreceptors and, thereby, influence rod photoreceptor survival. *Ire1*^*−/−*^ and *Xbp-1s*^*−/−*^ mice develop widespread developmental defects and undergo early embryonic death^28,29^. In contrast, *Atf6*^*−/−*^ mice are viable and have normal retinal morphology and function at birth and up to 3-month-old, but aged *Atf6*^*−/−*^ mice develop rod and cone dysfunction and retinal degeneration (18-month-old)^30^. Here, we test the role of *Atf6* in regulating rhodopsin protein levels and influencing retinal degeneration in P23H-KI mice by crossing *Atf6*^*−/−*^ with P23H-KI animals^23^.

## Results

### Loss of *Atf6* leads to impaired clearance of rhodopsin protein and hyperactivation of the IRE1-XBP-1s signaling pathway in early age *Rho*^*+/P23H*^ mice

Previously, we found that P23H rhodopsin protein was rapidly degraded in photoreceptors of P23H-KI mice at early age (P15)^22^. We also found that chemical-genetic activation of *Atf6* signaling pathways promoted P23H rhodopsin protein degradation in heterologous HEK293 cells, while sparing wild-type rhodopsin protein^22,27^. To investigate if *Atf6* is important for P23H rhodopsin protein degradation in photoreceptors, we examined retinas of *Rho*^+*/P23H*^ mice bred with *Atf6*^*−/−*^ mice.

**Figure 1:**
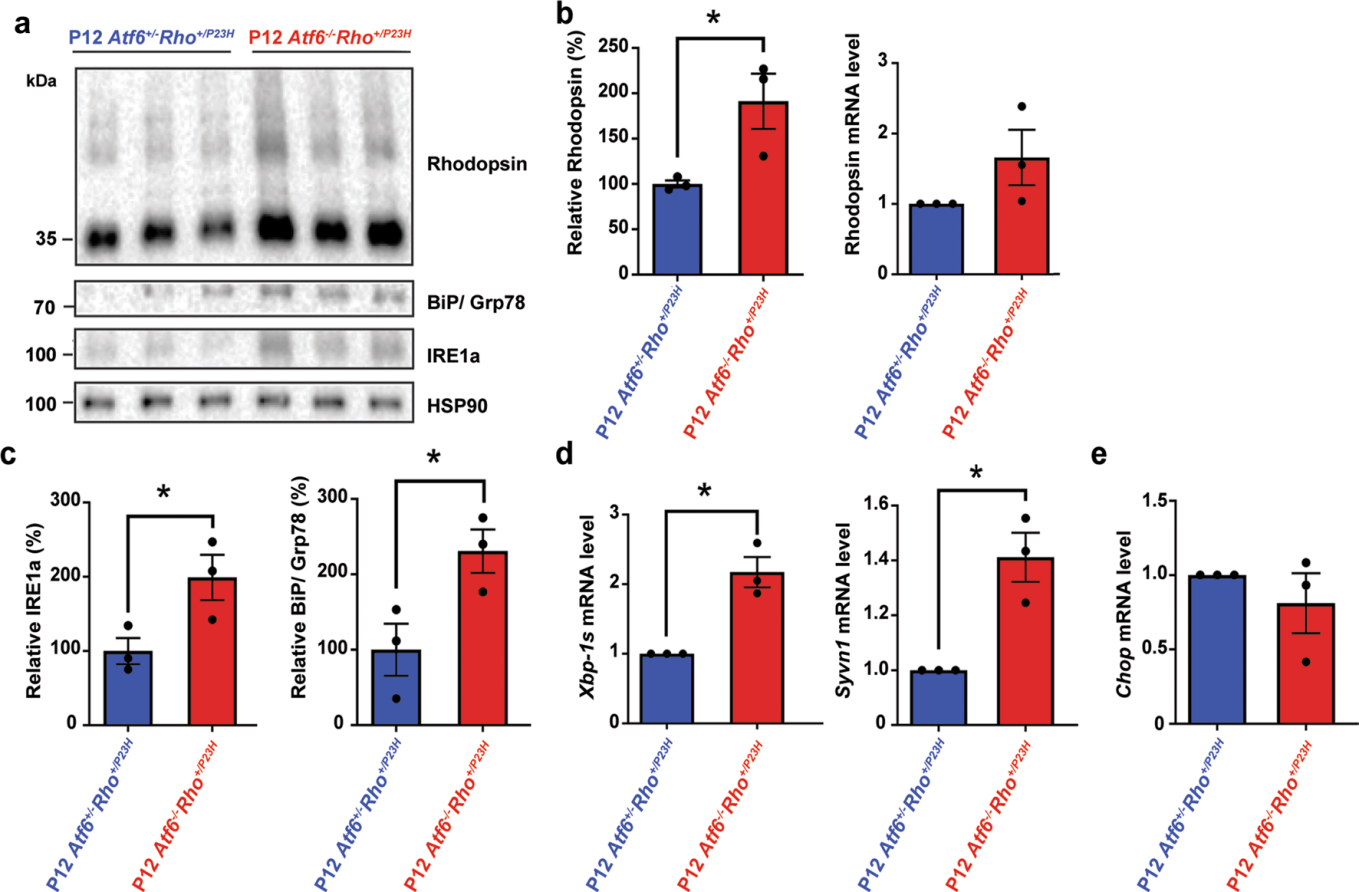
Elevated rhodopsin protein levels and increased IRE1 signaling activity in Rho + /P23H mice in the absence of *Atf6* in P12. (**a**) Retinas were collected at P12 and processed. Total rhodopsin, IRE1a, and BiP/Grp78 from *Atf6*^+*/−*^*Rho*^+*/P23H*^ and *Atf6*^*−/−*^*Rho*^+*/P23H*^ retinas were detected by immunoblotting and quantified. HSP90 served as loading control. (**b**) Total rhodopsin protein levels and mRNA levels were quantified in the retinas of *Atf6*^+*/−*^*Rho*^+*/P23H*^ (n = 3) and *Atf6*^*−/−*^*Rho*^+*/P23H*^ (n = 3) mice. (**c**) IRE1a and BiP/Grp78 protein levels were quantified in the retinas of *Atf6*^+*/−*^*Rho*^+*/P23H*^ and *Atf6*^*−/−*^*Rho*^+*/P23H*^ mice normalized to HSP90. Full-length blots are presented in Supplementary Fig. S2. (**d**) *Xbp-1s* and *Syvn1* mRNA levels in *Atf6*^*−/−*^*Rho*^+*/P23H*^ retinas (n = 3) were measured by real-time quantitative PCR and normalized to mRNA levels in *Atf6*^+*/−*^*Rho*^+*/P23H*^ retinas (n = 3). (**e**) *Chop* mRNA levels in both genotypes (n = 3) were measured by real-time quantitative PCR. Data are shown as mean ± SEM, three sets of independent experiments were performed. Data were analyzed by Student’s t-test and showed significance at *P < *0.05.*

First, we examined steady-state levels of rhodopsin in retinal protein lysates collected from *Atf6*^+*/−*^*Rho*^+*/P23H*^ and *Atf6*^*−/−*^*Rho*^+*/P23H*^ at P12, an age before morphological defects in photoreceptors or retinal degeneration emerges in *Rho*^+*/P23H*^ mice^22^. We saw significantly increased rhodopsin protein levels in *Atf6*^*−/−*^* Rho*^+*/P23H*^ retinas compared to *Atf6*^+*/−*^* Rho*^+*/P23H*^ retinas (191% increase, *P* = *0.04,* Fig. 1a,b). This finding demonstrates that *Atf6* is necessary for rhodopsin protein homeostasis in *Rho*^+*/P23H*^ mice retinas. *Rhodopsin* mRNA levels were not significantly different between *Atf6*^*−/−*^* Rho*^+*/P23H*^ and *Atf6*^+*/−*^* Rho*^+*/P23H*^ retinas (*P* = *0.5*, Fig. 1b) indicating that the differences seen in rhodopsin protein levels were not due to transcriptional differences. These findings support a role for *Atf6* in maintaining rhodopsin protein homeostasis in the retina via post-transcriptional mechanisms such as regulating its protein degradation.

Previously, we found that the IRE1-XBP-1 pathway was activated in *Rho*^+*/P23H*^ mice retinas and promoted P23H rhodopsin protein degradation in vitro^22,26^. To determine how loss of *Atf6* affected IRE1-XBP-1 in *Rho*^+*/P23H*^ mice, we examined biochemical and molecular markers of IRE1-XBP-1 activity in retinal lysates of P12 *Atf6*^+*/−*^* Rho*^+*/P23H*^ and *Atf6*^*−/−*^*Rho*^+*/P23H*^ mice. Interestingly, *Atf6*^*−/−*^*Rho*^+*/P23H*^ retinas showed significantly increased IRE1a and binding immunoglobulin protein/78-kDa glucose-regulated protein (BiP/Grp78) chaperone protein expression compared to *Atf6*^+*/−*^*Rho*^+*/P23H*^ retinas (*P* = *0.04*, Fig. 1c). To further investigate if IRE1 signaling pathway was increased in these retinas, we measured the mRNA levels of *Xbp-1s* (i.e. downstream target of IRE1 pathway) and ERAD-associated E3 ubiquitin-protein ligase HRD1/Synoviolin 1 (*Hrd1/Syvn1)* mRNA levels, an ER-associated protein degradation gene transcriptionally regulated by XBP-1s^12^. We found significant increase in the mRNA levels of *Xbp-1s* (*P* = *0.03*) and an Xbp-1s target gene, *Syvn1,* (*P* = *0.04*) in *Atf6*^*−/−*^*Rho*^+*/P23H*^ retinas compared to *Atf6*^+*/−*^*Rho*^+*/P23H*^ retinas (Fig. 1d). These findings provide evidence that IRE1 is hyper-activated in *Rho*^+*/P23H*^ retina in the absence of *Atf6* at this age.

By contrast to the changes observed in IRE1 pathway markers, *Chop* mRNA levels showed no significant differences between *Atf6*^+*/−*^*Rho*^+*/P23H*^ and *Atf6*^*−/−*^*Rho*^+*/P23H*^ mice (Fig. 1e) consistent with prior studies showing no induction of CHOP in mice expressing P23H rhodopsin^31^. In summary, we find that loss of *Atf6* in *Rho*^+*/P23H*^ mice leads to rhodopsin protein build-up in the retina at an early age, concomitant with increased activation of the IRE1 pathway.

### No gross changes in retinal histology in the absence of *Atf6* in young *Rho*^*+/P23H*^ retinas

**Figure 2:**
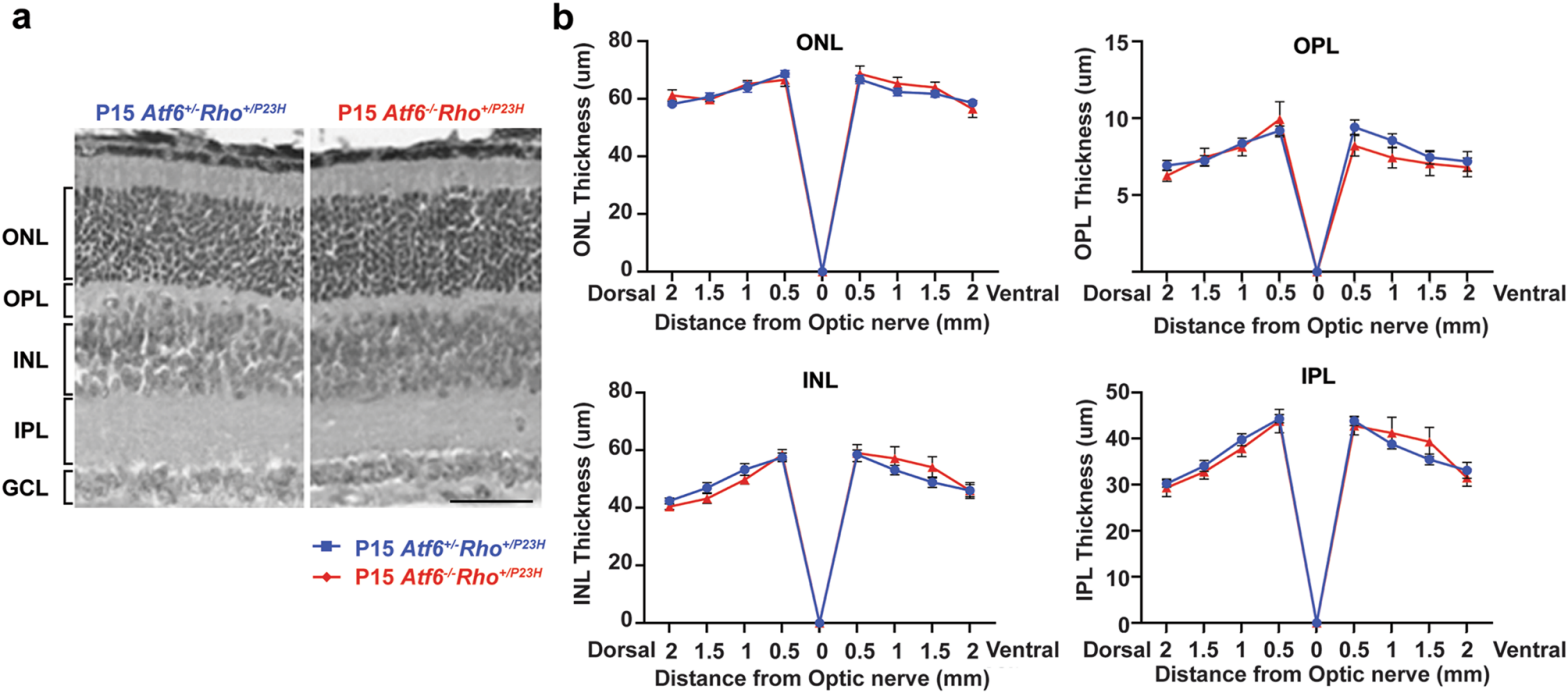
Quantitative spider plot analysis of P15 *Atf6*^+*/−*^*Rho*^+*/P23H*^ and *Atf6*^*−/−*^*Rho*^+*/P23H*^ retinas. (**a**) Light micrographs taken from vertical cryostat sections processed for H&E staining in P15 *Atf6*^*+/−*^*Rho*^+*/P23H*^ and *Atf6*^*−/−*^*Rho*^+*/P23H*^ retinas. (**b**) The retinal layers of H&E-stained retinal sections through the optic nerve (0) were measured at 8 locations around the retina, four each in the dorsal and ventral hemispheres. Retinal layer thickness between the two genotypes was not significantly different (n = 3–5, Data represents mean ± SEM, Two-way ANOVA, P > 0.5). *ONL* outer nuclear layer; *OPL* outer plexiform layer; *INL* inner nuclear layer; *IPL* inner plexiform layer. Scale bar = 50 μm.

Overexpression of rhodopsin causes photoreceptor cell death and induces retinal degeneration in transgenic animals expressing wild-type rhodopsin or P23H rhodopsin^32–34^. With that in mind, we asked if the increased steady-state rhodopsin protein levels in *Rho*^+*/P23H*^ mice lacking *Atf6* corresponded with photoreceptor cell loss (Fig. 1a). We performed histologic studies to see if photoreceptors or retinal lamination was impacted. A previously published paper has shown that *Rho*^+*/P23H*^ retinas have scattered pyknotic nuclei by P15^22^, with *Rho*^+*/P23H*^ mice exhibiting a slight reduction in the ONL thickness. Furthermore, the outer segments and inner segments of rod photoreceptors were shorter compared to the wild-type mice. In both *Atf6*^+*/−*^*Rho*^+*/P23H*^ and *Atf6*^*−/−*^*Rho*^+*/P23H*^ mice, we observed scattered and disorganized nuclei in the ONL ofP15 mice. In addition, the thickness of the ONL, outer plexiform layer (OPL), inner nuclear (INL), and inner plexiform layer (IPL) appeared similar (Fig. 2a,b) in retinas (*P* > *0.05*, two-way ANOVA analysis). Therefore, the ~ 2 × increase in rhodopsin protein levels found in the absence of *Atf6* did not lead to detectable changes in the thickness of photoreceptor ONL or overall retinal anatomy at this age. These findings demonstrate that *Rho*^+*/P23H*^ mice can tolerate increased amounts of rhodopsin protein when *Atf6* is lost, at least at this young age.

### Rhodopsin protein levels stabilize in intermediate age of *Rho*^*+/P23H*^ retina in the absence of *Atf6*

**Figure 3:**
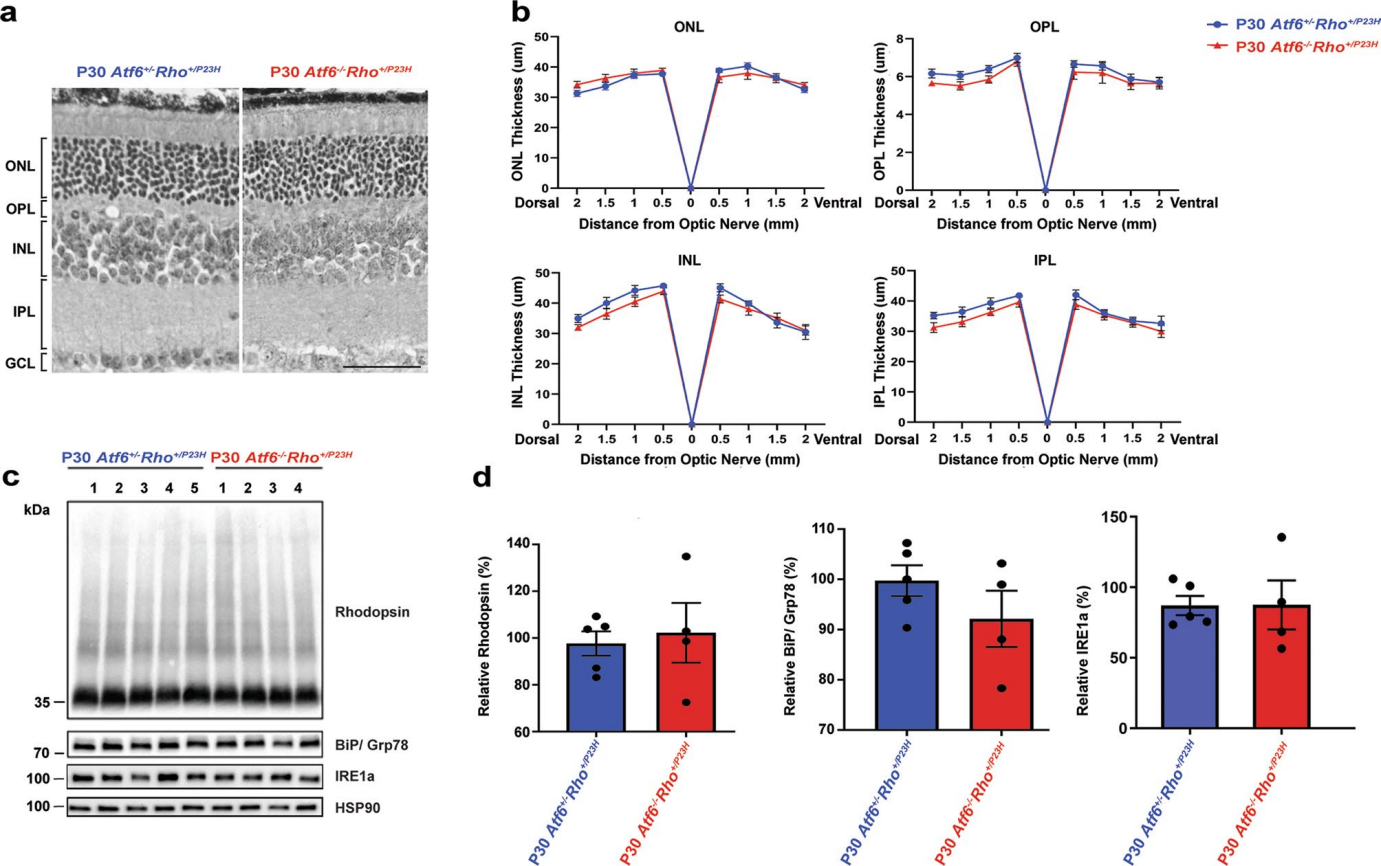
Rhodopsin protein levels in Rho + /P23H in the absence of *Atf6* at P30. (**a**) Light micrographs taken from vertical cryostat sections processed for H&E staining in P30 *Atf6*^*+/−*^*Rho*^*+/P23H*^ and *Atf6*^*−/−*^*Rho*^*+/P23H*^ retinas. (**b**) The retinal layers of H&E-stained retinal sections through the optic nerve (0) were measured at 8 locations around the retina, four each in the dorsal and ventral hemispheres. Quantification of retinal layer thickness between two genotypes showed no significant difference (n = 3–5, Data represents mean ± SEM, Two-way ANOVA, p > 0.5). *ONL* outer nuclear layer; *OPL* outer plexiform layer; *INL* inner nuclear layer; *IPL* inner plexiform layer. Scale bar = 50 μm. (**c**) Total rhodopsin, BiP/Grp78, and IRE1a from *Atf6*^*+/−*^*Rho*^*+/P23H*^ (n = 5) and *Atf6*^*−/−*^*Rho*^*+/P23H*^ (n = 4) retinas were detected by immunoblotting and quantified. HSP90 served as the loading control. Full-length blots are presented in Supplementary Fig. S3. (**d**) Rhodopsin, BiP/Grp78, and IRE1a protein levels were quantified and normalized to HSP90 in *Atf6*^*−/−*^*Rho*^*+/P23H*^ retinas compared to *Atf6*^*+/−*^*Rho*^*+/P23H*^ retinas. Data are shown as mean ± SEM, four to five sets of independent experiments were performed. Data were analyzed by Student’s t-test and showed significance at *P < 0.05.

Next, we examined the retina in older *Atf6*^+*/−*^*Rho*^+*/P23H*^ and *Atf6*^*−/−*^*Rho*^+*/P23H*^ mice. At P30, the thickness of retinal layers between *Atf6*^+*/−*^*Rho*^+*/P23H*^ and *Atf6*^*−/−*^*Rho*^+*/P23H*^ showed no significant difference (*P* > *0.05*, two-way ANOVA analysis, Fig. 3a,b). In both *Atf6*^+*/−*^*Rho*^+*/P23H*^ and *Atf6*^*−/−*^*Rho*^+*/P23H*^ mice, we observed scattered and disorganized nuclei in the ONL. To investigate if absence of *Atf6* altered rhodopsin protein levels in P23H retina at this age, we performed immunoblot analyses on the retinas of *Atf6*^+*/−*^*Rho*^+*/P23H*^ and *Atf6*^*−/−*^*Rho*^+*/P23H*^ P30 mice. In contrast to the increase in rhodopsin protein levels observed in younger mice (P12), retinal protein lysates of *Atf6*^*−/−*^*Rho*^+*/P23H*^ showed no significant difference in rhodopsin, BiP/Grp78, and IRE1a protein expression compared to *Atf6*^+*/−*^*Rho*^+*/P23H*^ at this age (Fig. 3c,d). We also found no significant increase in the mRNA levels of *Xbp-1s* in *Atf6*^*−/−*^*Rho*^+*/P23H*^ retinas compared to *Atf6*^+*/−*^*Rho*^+*/P23H*^ retinas (*P* > *0.05,* data not shown). These results show that loss of A*tf6* does not alter rhodopsin protein levels or retinal anatomy in *Rho*^+*/P23H*^ mice by this age. We propose that the equalization of rhodopsin protein levels in *Atf6*^*−/−*^* Rho*^+*/P23H*^ mice to levels seen in *Atf6*^+*/−*^*Rho*^+*/P23H*^ mice may be a result of the hyperactivation of IRE1-XBP-1s signaling observed in younger mice.

### Increased retinal degeneration in the absence of *Atf6* in *Rho*^+*/P23H*^ retina in older mice

**Figure 4:**
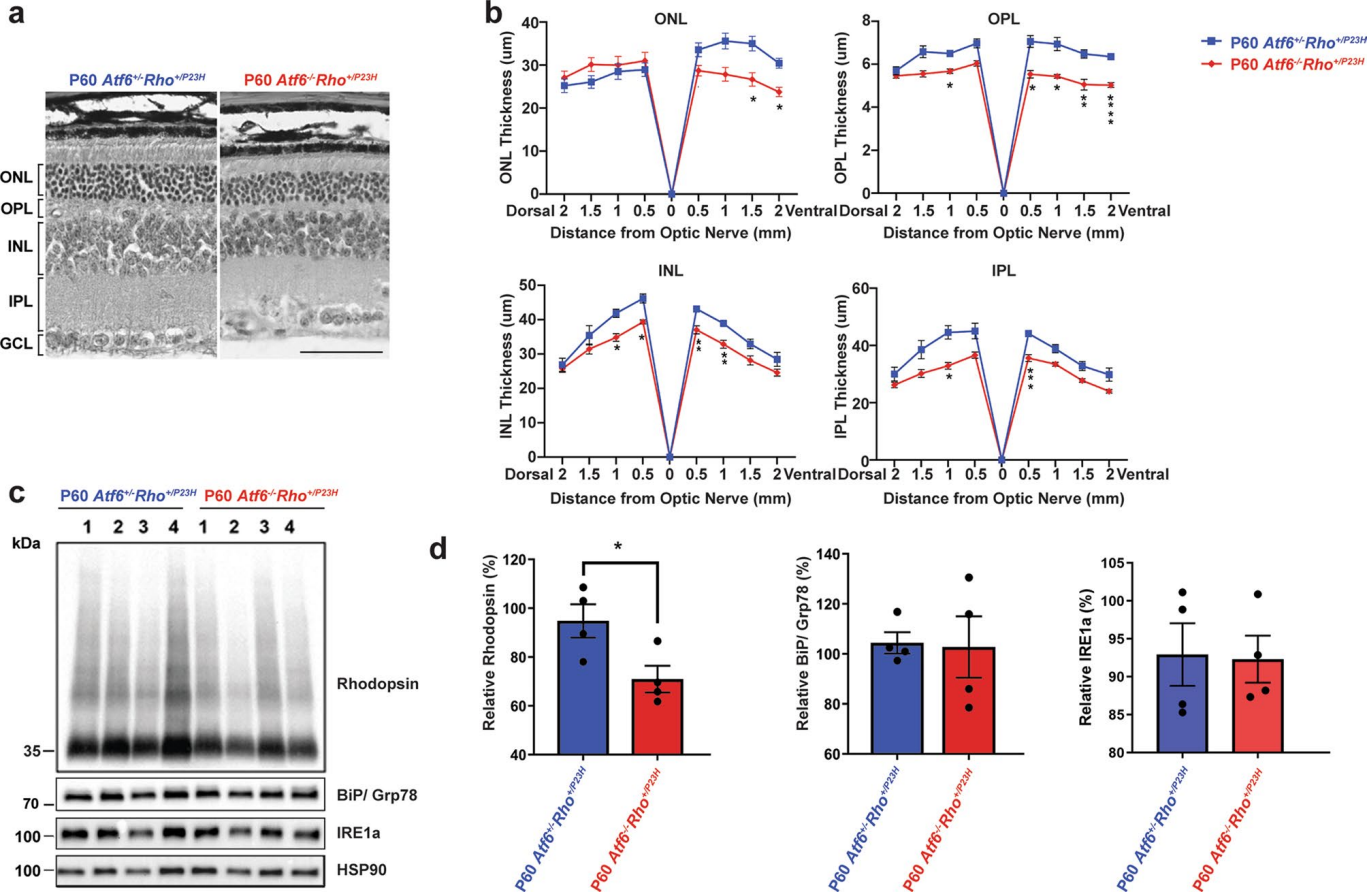
Loss of rhodopsin protein and attenuation of retinal lamina in Rho + /P23H in the absence of *Atf6* at P60. (**a**) Light micrographs taken from vertical cryostat sections processed for H&E staining in P60 *Atf6*^*+/−*^*Rho*^*+/P23H*^ and *Atf6*^*−/−*^*Rho*^*+/P23H*^ retinas. (**b**) The retinal layers of H&E-stained retinal sections through the optic nerve (0) were measured at 8 locations around the retina, four each in the dorsal and ventral hemispheres. The ventral retinal layers are significantly reduced compared to dorsal part of the retinas in *Atf6*^*−/−*^*Rho*^*+/P23H*^ compared to *Atf6*^*+/−*^*Rho*^*+/P23H*^ retinas at P60 (n = 3–5, Data represents mean ± SEM, Two-way ANOVA, *P < 0.05, **P < 0.005, ***P < 0.0005, ****P < 0.0001). *ONL* outer nuclear layer; *OPL* outer plexiform layer; *INL* inner nuclear layer; *IPL* inner plexiform layer. Scale bar = 50 μm. (**c**) Total rhodopsin, BiP/Grp78, and IRE1a from *Atf6*^*+/−*^*Rho*^*+/P23H*^ (n = 4) and *Atf6*^*−/−*^*Rho*^*+/P23H*^ (n = 4) retinas were detected by immunoblotting and quantified. HSP90 served as the loading control. Full-length blots are presented in Supplementary Fig. S4. (**d**) Rhodopsin, BiP/Grp78, and IRE1a protein levels were quantified in the retinas of *Atf6*^*+/−*^*Rho*^*+/P23H*^ and *Atf6*^*−/−*^*Rho*^*+/P23H*^ and normalized to HSP90. Reduction of rhodopsin protein levels was observed in *Atf6*^*−/−*^*Rho*^*+/P23H*^ compared to *Atf6*^*+/−*^*Rho*^*+/P23H*^ retinas while there were no changes in BiP/Grp78 protein levels in both genotypes. Data are shown as mean ± SEM, four sets of independent experiments were performed. Data were analyzed by Student’s t-test and showed significance at *P < 0.05.

Last, we examined *Rho*^+*/P23H*^ mice lacking *Atf6* at an older timepoint—P60. In contrast to P15 and P30 mice, morphological analysis of P60 retinas revealed significantly thinner ONL, OPL, INL, and IPL in *Atf6*^*−/−*^*Rho*^+*/P23H*^ when compared to A*tf6*^+*/−*^*Rho*^+*/P23H*^ retinas (Fig. 4a). Furthermore, the P60 *Atf6*^*−/−*^*Rho*^+*/P23H*^ showed shortening of outer segments and inner segments of photoreceptors in the ONL as previously described^22,23^. In the ONL, the ventral retinal ONL appeared to be selectively degenerated while the dorsal ONL was preserved in *Atf6*^*−/−*^*Rho*^+*/P23H*^ retinas (Fig. 4b; *P < 0.05; **P < 0.005, ***P < 0.0005, ****P < *0.0001*). Consistent with the loss of photoreceptors, we observed a reduction in total rhodopsin protein levels in *Atf6*^*−/−*^*Rho*^+*/P23H*^ retinas compared to *Atf6*^+*/−*^*Rho*^+*/P23H*^ retinas (*P* = *0.03,* Fig. 4c,d). Furthermore, no differences were observed in BiP/Grp78 or IRE1a levels at this age (Fig. 4c,d). These findings demonstrate that, by P60, loss of *Atf6* leads to increased retinal degeneration in *Rho*^+*/P23H*^ mice. We speculate that in the absence of *Atf6*, the duration and intensity of ER stress overwhelms the capacity of IRE1 to support ER homeostasis, leading to increased cell death at later ages of *Atf6*^*−/−*^*Rho*^+*/P23H*^ mice^35,36^.

### Assessment of scotopic and photopic function with ERGs

**Figure 5:**
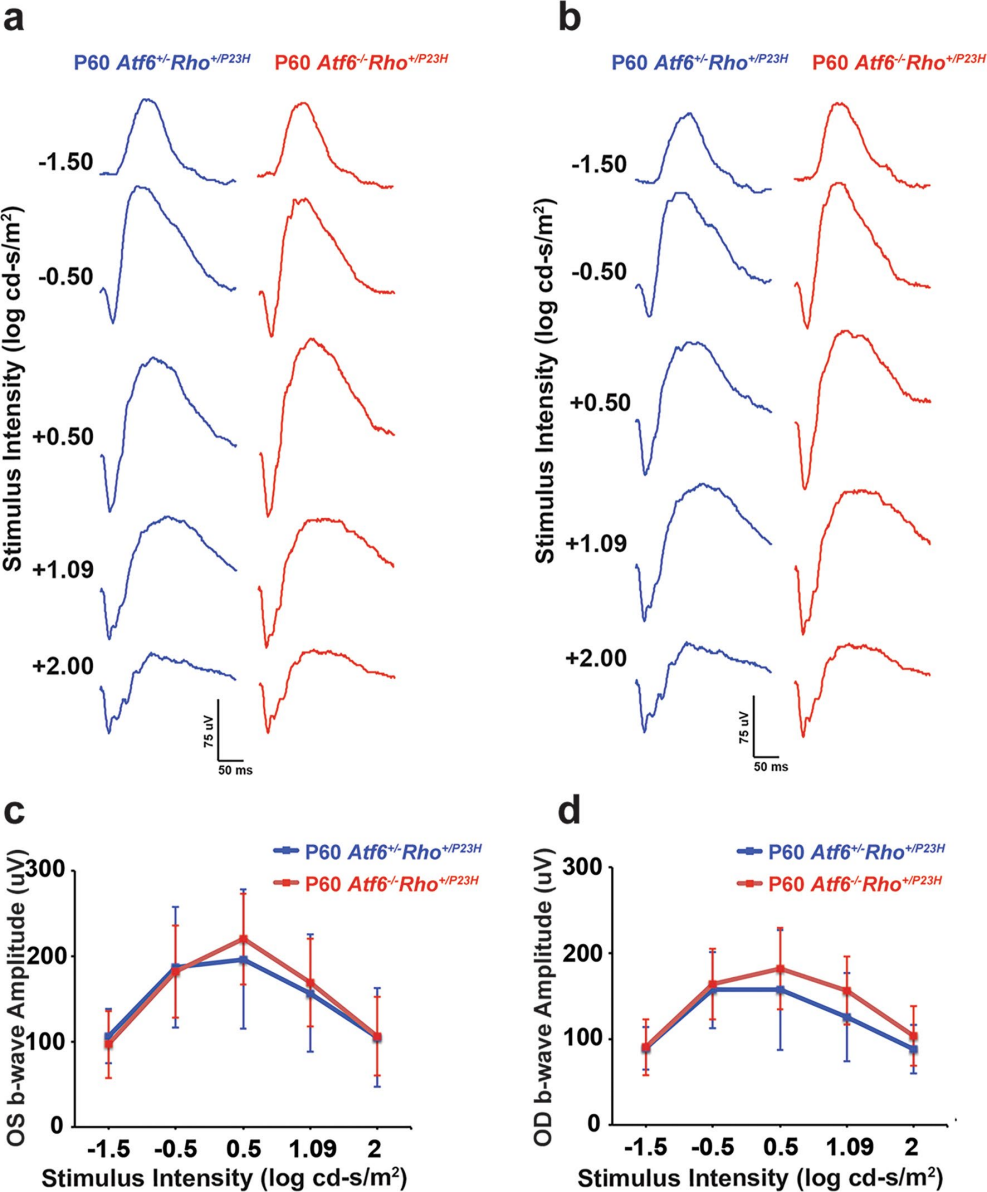
Scotopic ERG recordings from *Atf6*^+*/−*^*Rho*^+*/P23H*^ and *Atf6*^*−/−*^*Rho*^+*/P23H*^ retinas. (**a**) Representative waveforms generated by scotopic intensity series (− 1.5 to 2 log cd s/m^2^ stimuli) for *Atf6*^+*/−*^*Rho*^+*/P23H*^ (blue) and *Atf6*^*−/−*^*Rho*^+*/P23H*^ (red) in OS retinas. (**b**) Representative waveforms generated by scotopic intensity series (− 1.5 to 2 log cd s/m^2^ stimuli) for A*tf6*^+*/−*^*Rho*^+*/P23H*^ (blue) and *Atf6*^*−/−*^*Rho*^+*/P23H*^ (red) in OD retinas. (**c**,**d**) In P60, the b-wave amplitudes of *Atf6*^+*/−*^*Rho*^+*/P23H*^ (blue) and *Atf6*^*−/−*^*Rho*^+*/P23H*^ (red) in across multiple intensities, ranging from − 1.5 to 2 log cd s/m^2^ showed no significant difference (n = 6, Data represents mean ± SEM, Two-way ANOVA, *p* > *0.5*).

Last, we evaluated rod and cone function in these P60 *Rho*^+*/P23H*^ mice lacking *Atf6*. For the visual response of rods, we measured scotopic (Fig. 5) responses at P60 in *Atf6*^+*/−*^*Rho*^+*/P23H*^ and *Atf6*^*−/−*^*Rho*^+*/P23H*^ of both oculus sinister (OS) and oculus dexter (OD) by full-field ERG^30^. First, the scotopic ERG was recorded, and the amplitudes of the b-wave were analyzed (Fig. 5). In addition, an example of waveforms of the scotopic ERG responses from P60 *Atf6*^+*/−*^*Rho*^+*/P23H*^ (blue) and A*tf6*^*−/−*^*Rho*^+*/P23H*^ (red) (Fig. 5a,b) retinas were generated. For the amplitudes of the resulting b-wave responses at all light intensities, we did not detect differences in scotopic rod response between P60 *Atf6*^+*/−*^*Rho*^+*/P23H*^ and P60 *Atf6*^*−/−*^*Rho*^+*/P23H*^ mice. Based on these data, the reduction of retinal layers in ventral retina observed in P60 in absence of *Atf6* in *Rho*^+*/P23H*^ retina were likely not detected with full-field ERG due to preservation of the dorsal ONL layer.

**Figure 6:**
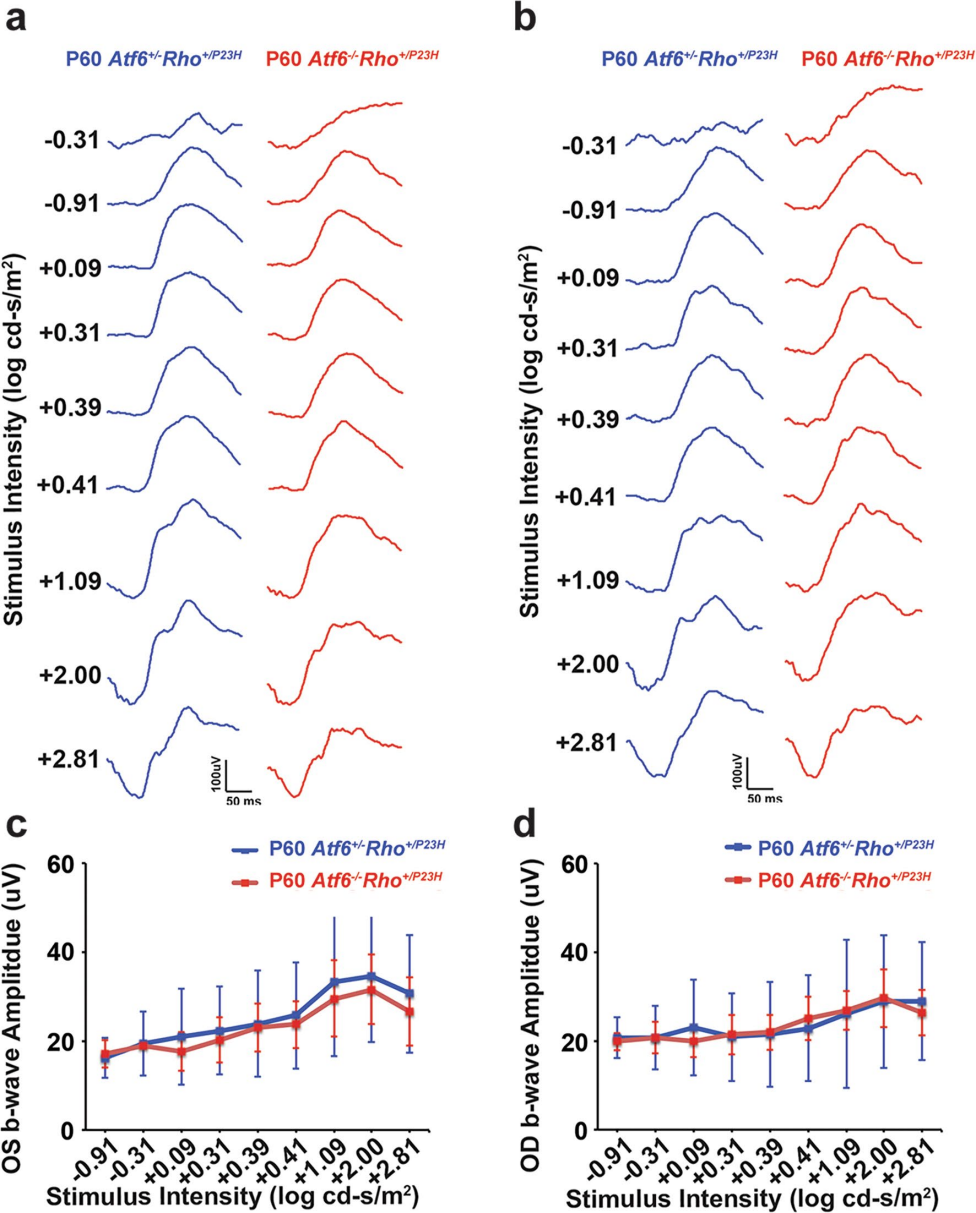
Photopic ERG recordings from *Atf6*^+*/−*^*Rho*^+*/P23H*^ and *Atf6*^*−/−*^*Rho*^+*/P23H*^ retinas. (**a**) Representative waveforms generated by photopic intensity series (− 0.31 to 2.81 log cd s/m^2^ stimuli) for *Atf6*^+*/−*^*Rho*^+*/P23H*^ (blue) and *Atf6*^*−/−*^*Rho*^+*/P23H*^ (red) in OS retinas. (**b**) Representative waveforms generated by photopic intensity series (− 0.31 to 2.81 log cd s/m^2^ stimuli) for *Atf6*^+*/−*^*Rho*^+*/P23H*^ (blue) and *Atf6*^*−/−*^*Rho*^+*/P23H*^ (red) in OD retinas. (**c**,**d**) In P60, the b-wave amplitudes of *Atf6*^+*/−*^*Rho*^+*/P23H*^ (blue) and *Atf6*^*−/−*^*Rho*^+*/P23H*^ (red) in across multiple intensities, ranging from − 0.31 to 2.81 log cd s/m^2^ stimuli showed no significant difference (n = 6, Data represents mean ± SEM, Two-way ANOVA, *p* > *0.5*).

The photopic ERGs also demonstrated no noticeable changes between P60 *Atf6*^+*/−*^*Rho*^+*/P23H*^ and P60 *Atf6*^*−/−*^*Rho*^+*/P23H*^ retinas (Fig. 6). An example of waveforms of the photopic ERG responses from *Atf6*^+*/−*^*Rho*^+*/P23H*^ (blue) and *Atf6*^*−/−*^*Rho*^+*/P23H*^ (red) (Fig. 6a,b) retinas are shown. For the amplitudes of the resulting b-wave responses at all light intensities, we were unable to detect differences in photopic cone response between *Atf6*^+*/−*^*Rho*^+*/P23H*^ and *Atf6*^*−/−*^*Rho*^+*/P23H*^ P60 mice. In addition, the pattern of waveforms between the two groups were consistent across all light intensities measured.

## Discussion

Many disease variants in the human *RHODOPSIN* gene found in RP patients introduce missense mutations in the rhodopsin polypeptide that cause rhodopsin protein misfolding, retention in the ER, and inability to bind to 11-cis-retinal^6,25,37,38^. These molecular defects instigate rod photoreceptor decline by incompletely understood mechanisms and, ultimately, lead to the clinical manifestations of RP. Currently, there is no cure for RP caused by these misfolded rhodopsin proteins. We previously found that chemical-genetic activation of the ATF6 signaling pathway significantly reduced protein levels of several misfolded RP rhodopsin variants, such as T17M, Y178C, C185R, D190G, and K296E rhodopsin, while sparing wild-type rhodopsin when expressed in heterologous HEK293 cells^39^. Furthermore, activation of ATF6 reduced misfolded P23H mutant rhodopsin protein levels (monomer, dimer, and multimers) in HEK293 cell in vitro^39^. Here, we investigated how ATF6 signaling affected P23H mutant rhodopsin protein in photoreceptors in vivo. We examined the steady-state levels of total rhodopsin protein in retinal samples collected from *Atf6*^+*/−*^*Rho*^+*/P23H*^ and *Atf6*^*−/−*^*Rho*^+*/P23H*^ mice. The rhodopsin protein species in these heterozygous *Rho*^+*/P23H*^ mice consist of wild-type and P23H rhodopsin. We found significantly more (nearly 2x) total rhodopsin protein in *Atf6*^*−/−*^* Rho*^+*/P23H*^ compared to *Atf6*^+*/−*^* Rho*^+*/P23H*^ while *rhodopsin* mRNA levels did not significantly change between these strains of mice at 12. These findings provide support that *Atf6* is important for rhodopsin protein quality control in rod photoreceptors, because in the *Atf6*^*−/−*^*Rho*^+*/P23H*^ mice, steady state rhodopsin protein levels increased almost 2x. ATF6 signaling likely ensures the efficient degradation of mutant P23H rhodopsin protein through transcriptional induction of factors involved in ER protein folding and ERAD^20,21,40,41^. Therefore, this model demonstrates that loss of *Atf6* leads to accumulation of P23H rhodopsin protein that contributes to the ~ 2 × increase in steady-state rhodopsin protein levels at early ages. Our findings may provide mechanistic insight into prior studies demonstrating a protective role for ATF6 activity in RP models. For example, in vivo intravitreal AAV injection of one of ATF6’s downstream targets, the BiP/Grp78 chaperone, into P23H rhodopsin transgenic rats improved ERG responses^42^. This protective response could arise from increased elimination of the P23H rhodopsin protein through increased BiP/Grp78 chaperone-mediated increase in ERAD. Taken together, these findings underscore the importance of *Atf6* plays in rhodopsin protein homeostasis in rods.

Variants in the human *ATF6* gene cause achromatopsia and cone-rod dystrophy carrying bi-allelic disease alleles^30,43–47^. Patients with these *ATF6* mutations showed malformation of the fovea, dysfunction of photoreceptors, and severe vision loss from infancy^30,45^. Furthermore, it is reported that abnormal retinal vasculature development may lead to malformation of the fovea^48,49^. However, none of these findings are apparent in young *Atf6*^*−/−*^ mice or in young *Atf6*^*−/−*^*Rho*^+*/P23H*^ mice^30^ (Supplemental Fig. S1). This difference may reflect a selective function for ATF6 in human cone and/or foveal development. For example, retinal organoids produced from the patients homozygous for *ATF6* disease alleles showed significant defects in cone photoreceptor development accompanied by reduction in cone gene expression which included all cone phototransduction genes (*CNGB3, CNGA3, PDE6C, PDE6H,* and *GNAT2*) and red and green cone opsin genes^50^. Although cones do not appear to be selectively compromised in *Atf6*^*−/−*^ mice or *Atf6*^*−/−*^*Rho*^+*/P23H*^ mice, the absence of *Atf6* does accelerate degeneration throughout the retina in *Rho*^+*/P23H*^ mice. This is consistent with *Atf6* expression in all retinal cell types, where it likely functions to ensure cell viability in the face of ER stress throughout life^47^.

In our study, we found that the IRE1 signaling pathway was hyper-activated in *Atf6*^*−/−*^*Rho*^+*/P23H*^ mice when rhodopsin steady-state levels were increased at young ages. We propose that this hyper-activity in IRE1 signaling reflects a compensatory response to loss of *Atf6*. Specifically, the loss of *Atf6* leads to reduced degradation of mutant rhodopsin protein in *Rho*^+*/P23H*^. In turn, this accumulation of misfolded rhodopsin hyper-activates the IRE1 signaling pathway to degrade the increased rhodopsin accumulating in P12 *Atf6*^*−/−*^* Rho*^+*/P23H*^. Consistent with a role for IRE1 signaling in rhodopsin degradation, we have previously demonstrated that the IRE1 signaling pathway of the UPR is selectively activated in photoreceptors of *Rho*^+*/P23H*^*ERAI*^+*/−*^ compared to *Rho*^+*/*+^*ERAI*^+*/−*^ mice in a study that used the ERAI mouse GFP reporter line to indicate IRE1-XBP-1 activation^22^. We found that induction of ERAD by IRE1 signaling leads to ubiquitination of P23H rhodopsin in photoreceptors in *Rho*^+*/P23H*^ mice^22^. This demonstrated that P23H rhodopsin is rapidly degraded by induction of ERAD in photoreceptors to eliminate misfolded rhodopsin from the ER in vivo. Furthermore, in a 2021 ARVO poster, Massoudi et al. selectively deleted the gene encoding IRE1a in rod photoreceptor in *Rho*^+*/P23H*^ mice and demonstrated that ablation of *Ire1a* in rod photoreceptors damaged retinal function and increased retinal degeneration in *Rho*^+*/P23H*^ mice^51^. Based on our previous and current study and the recent report by Massoudi et al. (2021), both ATF6 and IRE1a protect against ER stress in photoreceptors in *Rho*^+*/P23H*^ mice. Our current study showed that the levels of *Xbp-1s* mRNA, BiP/Grp78 protein, and other transcriptional targets were significantly increased in the retinas of *Atf6*^*−/−*^* Rho*^+*/P23H*^ mice compared to *Atf6*^+*/−*^*Rho*^+*/P23H*^ mice at early age. Many of XBP-1’s target genes encode components of the ERAD pathway, and these genes have been found to be upregulated in the retinas of *Rho*^+*/P23H*^ mice^12,13,22^. These findings suggest that degradation of P23H rhodopsin via downstream transcriptional activity of the IRE1-XBP-1s pathway and, consequently, ERAD, both work to alleviate ER stress caused by the accumulation of misfolded rhodopsin. Our model is further supported by the findings that E3 ubiquitin ligases, SORDD1/2, was able to facilitate degradation of Rh1^P37H^ (the Drosophila equivalent of P23H rhodopsin) at larval and earlier stages of growth to allow for development of healthy adult eyes. Furthermore, SORDD1/2 and HRD1/SYVN1 were also able to prevent retinal degeneration in Drosophila with the G69D (glycine to aspartic acid at amino acid residue 69) rhodopsin mutation^52^. The lack of *Atf6* in *Rho*^+*/P23H*^ mice may initially increase the ability of E3 ubiquitin ligases downstream of IRE1-XBP-1s-ERAD to target misfolded rhodopsin in early stages of life. In contrast, *Chop* mRNA levels (an ER stress gene induced by PERK pathway)^22,53^ were not affected between *Atf6*^+*/−*^* Rho*^+*/P23H*^ and *Atf6*^*−/−*^*Rho*^+*/P23H*^ mice, which is consistent with previous studies showing that *Chop* was not induced during retinal degeneration in P23H rhodopsin mice and that the loss of CHOP had no impact on retinal degeneration based on histology or ERG^31,54^. Activation of PERK signaling also did not lead to greater reduction in rhodopsin protein levels in WT or P23H mice^27^.

There are several lines of evidence suggesting alterations of other degradation systems in *Atf6*^*−/−*^*Rho*^+*/P23H*^ mice. We have previously reported in cell culture models that IRE1 relies on functioning proteasomes and lysosomes to degrade the mutated, misfolded rhodopsin^27^. Yao et al. (2018) also reported that P23H mice experience increase in autophagy secondary to ER stress, which leads to proteasome insufficiency and increase retinal degeneration. In contrast, genetic or pharmacologic inhibition of autophagy reduced retinal degeneration and improved proteasome levels^55^. Modulating the ratio between autophagy and proteasome activity (A:P) also helped to improve photoreceptor survival^56^. The authors demonstrated that normalizing the A:P ratio, either by improving folding of P23H rhodopsin or increasing proteasome activity to keep autophagy pathways down, increased photoreceptor survival and preserved retinal function. Taken together, we suggest that autophagy activity is increased as a result of the loss of *Atf6.*

We found increased retinal degeneration and diminished rhodopsin protein levels in P60 *Atf6*^*−/−*^*Rho*^+*/P23H*^ retinas compared to P60 *Atf6*^+*/−*^*Rho*^+*/P23H*^. Furthermore, we found that the thickness of retinal layers including ONL, OPL, INL, and IPL were also significantly lower in the ventral part of the *Atf6*^*−/−*^*Rho*^+*/P23H*^ retina compared to *Atf6*^+*/−*^*Rho*^+*/P23H*^. The reduction of ONL in the ventral part of the retina is consistent with previous histological data but the thickness of other retinal layers was not measured previously^22–24^. In *Rho*^+*/P23H*^ mice, approximately half of the rod photoreceptor cells had disappeared between P14-P40 when compared to *Rho*^+*/*+^ retina, which showed no reduction of rod photoreceptors between P40 and P63 as described in previous studies^22–24^. Our data demonstrate that by P60, loss of *Atf6* accelerates retinal degeneration in *Rho*^+*/P23H*^ mice. Why does loss of *Atf6* increase retinal degeneration in *Rho*^+*/P23H*^ mice at P60, while not affecting younger animals? Our previous study showed that early wave of photoreceptor cell death and peak induction of the IRE1 reporter occur during the first postnatal month in *Rho*^+*/P23H*^ mice^22^. The activation of IRE1 in *Rho*^+*/P23H*^ is maintained throughout life to regulate proteostatic balance to remove P23H rhodopsin^22^. We propose that hyperactivation of IRE1 (as seen in the younger animals) restored rhodopsin protein homeostasis in the absence of *Atf6* beginning at P12 (i.e., early stage), so that P30 *Rho*^+*/P23H*^ retinas looked indistinguishable. Why can’t IRE1 hyperactivation keep rhodopsin and retina healthy at P60? We propose that the capacity of IRE1 to support ER homeostasis may ultimately be overwhelmed in the absence of *Atf6*, leading to increased rod photoreceptor cell death, and reduction of rhodopsin protein levels at later ages of *Atf6*^*−/−*^*Rho*^+*/P23H*^ mice^35,36^. The ongoing photoreceptor cell death in RP retina likely causes widespread ER stress from oxidative damage, mitochondrial dysfunction, and other metabolic degenerative mechanisms^57,58^. Other sources of ER stress arising in the degenerating retina include damaged lipids, proteins, carbohydrates, enzymes, and DNA in photoreceptor cells, which ultimately results in further photoreceptor cell death through lipid peroxidation^59^. Thus, P23H rhodopsin-induced cell damage in addition to P23H rhodopsin protein itself could elicit too much ER stress, overwhelming the proteostatic balance maintained by the IRE1 in in the *Atf6*^*−/−*^*Rho*^+*/P23H*^ mice.

Here, we observed no detectable difference in the function of rods and cones between P60 *Atf6*^+*/−*^*Rho*^+*/P23H*^ and *P60 Atf6*^*−/−*^*Rho*^+*/P23H*^ mice. Although, we observed a reduction of ONL and other retinal layers in *Atf6*^*−/−*^*Rho*^+*/P23H*^ mice compared to *Atf6*^+*/−*^*Rho*^+*/P23H*^ mice, no significant difference was noted in amplitude of either the scotopic or photopic b-wave in the strains of mice. Why did the reduction of retinal layers in *Atf6*^*−/−*^*Rho*^+*/P23H*^ mice compared to *Atf6*^+*/−*^*Rho*^+*/P23H*^ mice show no functional changes? We propose that the full-field flash ERG is relatively insensitive to detect smaller defects^60,61^ because it represents the global retinal function via summed electrical response of the whole retina excited by a flash of light^62^. Thus, the reduction of retinal layers in ventral retina observed in P60 in the absence of *Atf6* in *Rho*^+*/P23H*^ retina was likely not detected with full-field ERG due to preservation of the dorsal ONL layer.

In recent years, numerous small molecules have been identified that activate or inhibit ATF6 or IRE1^27,63–68^. Agonists of IRE1 signaling include Type 1 IRE1 kinase inhibitors, which allosterically activate the RNAse function of IRE1, and IRE1 activators, which activate both RNAse and kinase function; however, these small molecule candidates (e.g. 474, IXA4, and IXA6), albeit showing no activation of IRE1-dependent cell death pathways, have yet to be fully tested for rhodopsin proteostatic properties^67,69^. By contrast, ATF6 agonists (e.g., AA147 and AA263) are effective in vivo and may have significant implications for amyloid related diseases and retinal development through ATF6 activation^53,69,70^. Furthermore, research from other groups similarly propose that BiP/Grp78, a prominent target of ATF6 upon ER stress, alleviates P23H RP symptoms^42^. We propose that ATF6 and IRE1-XBP-1 small molecule agonists are promising agents for further RP clinical studies if their rhodopsin proteostatic properties can be *shown *in vivo.

## Methods

### Animals

Transgenic *Atf6*^+*/*+^ and *Atf6*^*−/−*^^30,41^ and Rho P23H-KI mice^23,24^ on a pure C57BL/6J background were used to generate *Atf6*^+*/−*^*Rho*^+*/P23H*^ and *Atf6*^*−/−*^*Rho*^+*/P23H*^ mice for the experiments. First, breeding pairs of *Atf6*^+*/*+^*Rho*^+*/P23H*^ and *Atf6*^*−/−*^*Rho*^+*/*+^ mice of C57BL/6J background were crossed to generate *Atf6*^+*/−*^*Rho*^+*/P23H*^. Breeding pairs of A*tf6*^*−/−*^*Rho*^+*/P23H*^ with *Atf6*^*−/−*^*Rho*^+*/*+^ mice of C57BL/6J background were crossed to generate *Atf6*^*−/−*^*Rho*^+*/P23H*^.

There are no reported differences in the phenotypes between *Atf6*^+*/*+^ and *Atf6*^+*/−*^ mice^30,41,42,44^. Injection of *Atf6*^+*/*+^, *Atf6*^+*/−*^, and *Atf6*^*−/−*^ mice with tunicamycin led to kidney and liver toxicity only in *Atf6*^*−/−*^ animals but not in *Atf6*^+*/*+^ or *Atf6*^+*/−*^ mice; in addition, our previous study has shown normal morphology and normal rhodopsin expression when comparing *Atf6*^+*/*+^ to *Atf6*^+*/−*^ mice^30^. All experiments used female or male *Atf6*^*−/−*^*Rho*^+*/P23H*^ mice in comparison to control littermates *Atf6*^+*/−*^*Rho*^+*/P23H*^, at the postnatal (P) days 12, 15, 30, and 60 (number (n) = 3 ~ 6 respectively for each stage). For retinal vasculature assessment in *Atf6*^*−/−*^ mice, female and male P30 *Atf6*^+*/*+^ and P30 *Atf6*^*−/−*^ mice (n = 3 animals per group) on a C57BL/6 J background were used as described in previous studies^30,41^. For all experiments, animals were kept in cyclic 12-h light/dark conditions with free access to food and water. All mouse care and experimental procedures in this study were approved and conducted in strict accordance with relevant guidelines and regulations by the Institutional Animal Care and Use Committee at the Stanford University and in compliance with the Association for Research in Vision and Ophthalmology Statement for the Use of Animals in Ophthalmic and Vision Research and the ARRIVE (Animal Research: Reporting of in Vivo Experiments) guidelines.

### Tissue preparation

The animals were euthanized by carbon dioxide euthanasia at P12, P15, P30, and P60. The eyes were enucleated for collection of retinal tissue. For secondary method, we performed cervical dislocation. The lens and the anterior segment were removed, and the eyecups were further dissected to collect whole retinal lysate for biochemistry or molecular biology, or eyecups were fixed in 4% paraformaldehyde in 0.1 M phosphate buffer (PB), for 60 min at 4 °C. After fixation, the eyecups were processed for hematoxylin and eosin (H&E) staining^71^ and cryostat sectioning. For cryostat sectioning, eyecups were transferred from 10% for 1 h to 20% for 1 h to 30% sucrose overnight at 4 °C, then eyecups were embedded in Optimal Cutting Temperature (OCT) medium (Tissue-Tek, Elkhart, IN), frozen in liquid nitrogen and subsequently vertically sectioned on a Leica cryostat (Leica Biosystems Inc, Buffalo Grove, IL) at a thickness of 20 μm. For wholemount retinal preparation, the retinas were isolated from the eyecups and dissected as wholemounts.

### H & E staining

The detail protocols for H & E staining in retinal layer was performed as previously published^71^. Three to five left eyecups from three to five animals (n = 3–5) were sectioned along the vertical meridian on a cryostat at a thickness of 20 μm. Sections were then collected on gelatin-coated slides for H&E staining. Slides were dipped in Harris hematoxylin for 1 min then they were washed in tap water and dehydrated in alcohol. Slides were then dipped in Eosin-Phloxyine for 30 s, then dehydrated in a series of 95% ethanol and 100% ethanol followed by 5 min in xylene, and mounted in Vectashield mounting medium (Vector Labs, Burlingame, CA).

### Immunoblotting analysis

The detail protocols for immunoblotting analysis were performed as previously published^27,45^. Three to five right retinas from three to five animals (n = 3–5) were lysed in lysis buffer (0.5 g/mL n-Dodecyl β-d-maltoside (Calbiochem EMD Bioscience, San Diego, CA) in PBS), protease inhibitor (Sigma-Aldrich, St. Louis, MO) and phosphatase inhibitor (Thermo Scientific, Rockford, IL). Protein concentrations of the total retinal lysates were determined by BCA protein assay (Pierce, Rockford, IL). Equal amounts of protein were applied onto 4–15% Mini-PROTEAN TGX precast gels (Bio-Rad, Hercules, CA) and analyzed by immunoblot. Antibodies B630N anti-rhodopsin 1:1000^39^ (gift of W.C. Smith, Gainesville, FL); anti-BiP/Grp78 at 1:1000^27^, anti-IRE1a at 1:1000^72^, and anti-HSP90 at 1:1000^22^ (GeneTex, Inc., Irvine, CA) were used. After overnight incubation with primary antibody in a 4 °C cold room, membranes were washed in TBS with 0.1% Tween-20, followed by incubation of a horseradish peroxidase-coupled secondary antibody (Cell Signaling, Danvers, MA). Immunoreactive bands were detected with the SuperSignal West chemiluminescent substrate (Pierce, Rockford, IL).

### Quantitative PCR analysis (qPCR)

The detail protocols for qPCR analysis were performed as previously published^44,45^. Three to five right retinas from three to five animals (n = 3–5) were lysed and total RNA was collected with a RNeasy mini kit (Qiagen, Germany) and mRNA was reverse transcribed with the iScript cDNA Synthesis Kit (Bio Rad, Hercules, CA). Primers that were used included^22,31^: mouse *Rhodopsin* mRNA, 5′-TTCACCACCACCCTCTACACATCAC-3′ and 5′-CGGAAGTTGCTCATCGGCTTG-3′; mouse *Xbp-1s* mRNA, 5′- GAGTCCGCAGCAGGTG-3′ and 5′-GTGTCAGAGTCCATGGGA-3′; mouse *Syvn1*, 5’- ACACACTACTGGATGCTGCC-3’ and 5’- GCTTCAGGAATTGGTGGGGA-3’; mouse *Chop*: 5′- ACGGAAACAGAGTGGTCAGTGC-3′ and 5′-CAGGAGGTGATGCCCACTGTTC-3′, and mouse *Rpl19*: 5′-ATGCCAACTCCCGTCAGCAG- 3′ and 5′- TCATCCTTCTCATCCAGGTCACC-3′. *Rpl19* mRNA levels were used as internal normalization standards for qPCR analysis as they were not altered by ER stress. qPCR conditions were 95 °C for 5 min; 95 °C for 10 s; 60 °C for 10 s; 72 °C for 10 s, with 50 cycles of amplification.

### Electroretinography (ERG) and quantification

Mice were dark adapted for 24 h prior to recordings. ERGs (Diagnosys LLC, Lowell, MA) were recorded from both eyes of *Atf6*^*−/−*^*Rho*^+*/P23H*^ (n = 6) mice and compared to ERGs from eyes of *Atf6*^+*/−*^*Rho*^+*/P23H*^ control littermates (n = 6) at P60 as described previously^30^. Mice were anaesthetized using a combination of ketamine (20 mg/kg; KETASET, Fort Dodge, IA, USA) and xylazine (5 mg/kg, X-Ject SA; Butler, Dublin, OH, USA) using similar procedures as our published protocols^30^. Under a dim red light, the pupils were dilated with Atropine sulfate ophthalmic solution 1% (Akorn Inc, Lake Forest, IL, USA). The recording electrodes attached to two gold wire rings were placed on the cornea of both eyes. The eye lubricant hypromellose ophthalmic gel, USP 2.5% (HUB pharmaceuticals, LLC, Rancho Cucamonga, CA, USA) was applied to keep the hydration and conductivity between the cornea and recording electrodes. The ground and reference electrodes were placed at the tail and tongue, respectively. The eyes were then given scotopic ERG responses (a series of white light flashes varying from -1.5 to 2 log cd s/m^2^). After 10 min of light adaptation, photopic ERG responses of -0.31 to 2.81 log cd s/m^2^ were recorded. The amplitudes for the resulting b-wave responses at the series of light flash intensity were plotted.

### Retinal vasculature staining in *Atf6*^*−/−*^ mice

For wholemount immunohistochemical staining, the same procedures described in our previous studies were used^30,71^. Three right retinas from three animals (n = 3) were used for wholemount staining. Wholemounts were treated with 1% Triton X-100 in 0.1 M PBS (40 min) before NDS (1 h), and antibody against isolectin B4-Alexa 488 (IB4, molecular probe, 1:200)^73^ was diluted in 0.5% Triton X-100 in 0.1 M PBS (48 h at 4 °C). After incubation, wholemounts were washed for 30 min with 0.1 M PB and cover slipped with Vectashield mounting medium.

### Equipment and settings

IB-4 staining wholemount (excitation 488, emission 552, 63 × 1.40 oil objective) images were acquired using a Leica SP8 DLS confocal microscope. Images were processed with the Leica application suite-X software (3.0.11.20652, Leica Mcirosystems—Dimension X × Y—local size 1024 × 1024 pixels, 8 Bit for superficial layer, intermediate layer, and deep layer; Dimension X × Y-local size 4753 × 4753 pixels, 8 Bit for entire wholemount images). For retinal vasculature assessment in *Atf6*^*−/−*^ mice, confocal micrographs of the wholemounts (n = 3, animals per group) were taken at the nerve fiber layer (superficial layer), at the IPL (intermediate layer), and at the OPL (deep layer) of the dorsal regions (1 mm away from optic disc) of the retina. At these regions, serial optical section (Dimension z, 2 µm intervals) was made using a confocal microscope. H & E staining sections (20 × objective) images under brightfield were acquired using a NanoZoomer 2.0-HT slide scanner NDP scan 2.5 and viewed using NDP view 2 (Hamamatsu Photonics). The Hamamatsu NanoZoomer uses 3-chip time-delay integration (TDI) sensor signal. Images were processed with the Image Lab Touch Software version 3.0 (Bio Rad). For retinal layer thickness measurement, thickness of the retina was measured at 0.5 mm intervals beginning from the optic nerve. For each retinal section, three measurements of the ONL, OPL, INL, and IPL thickness were taken for each section (fields covering 350 µm × 350 µm), spaced approximately 100 µm apart, which were then averaged^74^. Layer thickness measurements were collected from three to five retinas from separate *Atf6*^+*/−*^*Rho*^+*/P23H*^ and *Atf6*^*−/−*^*Rho*^+*/P23H*^ mice. The results were plotted as a spider plot with distance from the optic nerve as the x-axis and thickness of retinal layer as the y-axis. Intensity of Immunoreactive bands in all blots were measured with National Institute of Health (NIH) Image J software version 1.50i. For presentation, all Photoshop (Adobe photoshop CC 2020) adjustments (brightness and contrast only) were carried out equally in each figure.

### Statistical analysis

All the statistics were expressed as mean ± standard error of the mean (SEM). Student’s t-test was used for comparison. Two-way ANOVA and Fisher's least significant difference procedure (LSD test) were used to examine the differences among the group of means. All the statistical tests were performed using GraphPad Prism Version 8.3.1. The difference between the means of separate experimental groups was considered statistically significant at P < 0.05.

## Supplementary Information

Supplementary Information.
