# Supplementary material for: ATF6 is required for efficient rhodopsin clearance and retinal homeostasis in the P23H rho retinitis pigmentosa mouse model
Source: Sci Rep. 2021 Aug 11;11:16356. doi: 10.1038/s41598-021-95895-7 (PMC8357971; doi:10.1038/s41598-021-95895-7)
Supplement: Supplementary file 1 — Supplementary Information. [file 41598_2021_95895_MOESM1_ESM.docx]

**ATF6 is Required for Efficient Rhodopsin Clearance and Retinal Homeostasis in the P23H Rho Retinitis Pigmentosa Mouse Model**

Eun-Jin Lee^1,2,3,4*^, Priscilla Chan^5*^, Leon Chea^1,2,3^, Kyle Kim^1,2,3^, Randal J. Kaufman^6^ & Jonathan H. Lin^1,2,3†^


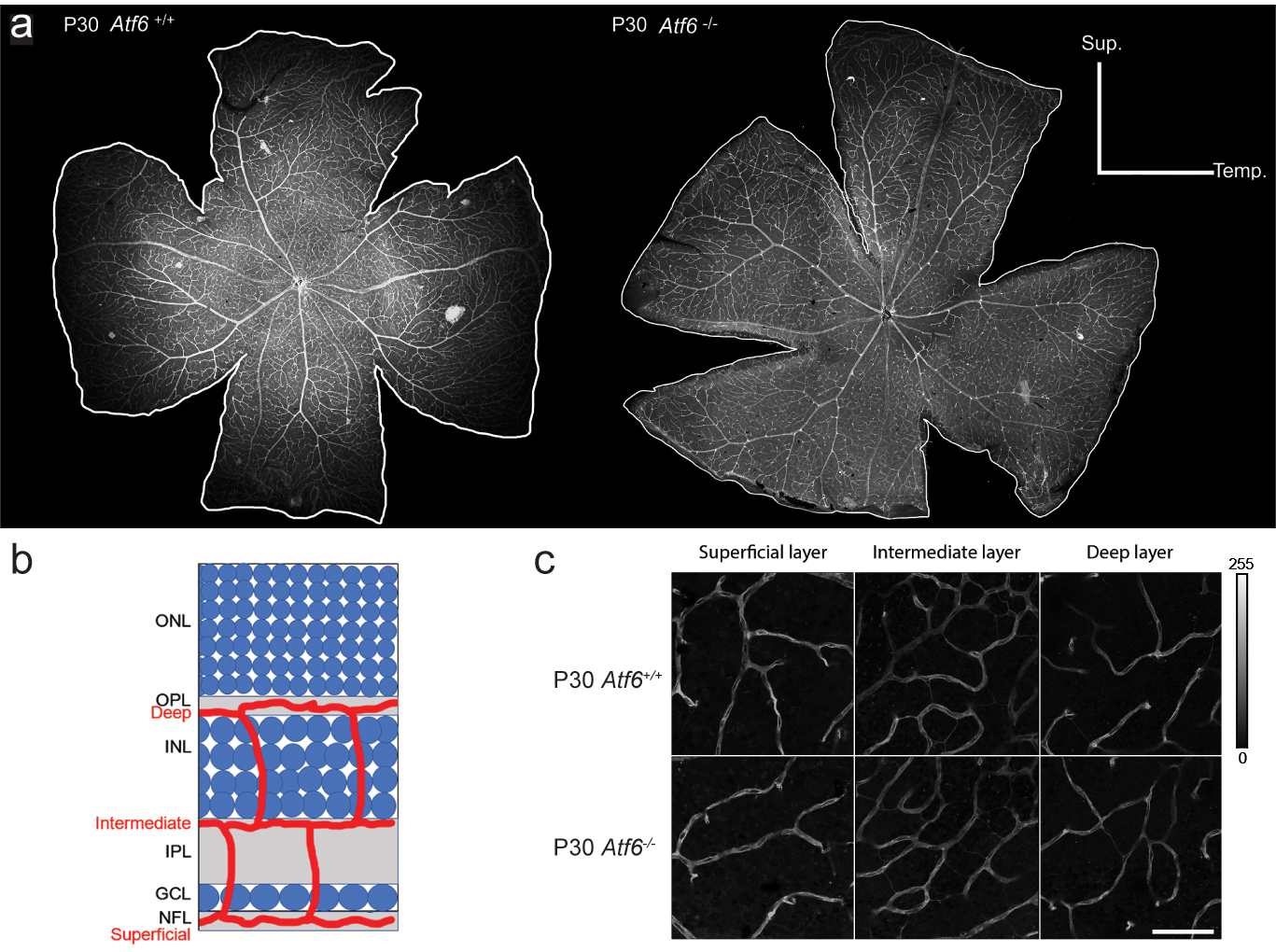


**Supplemental Figure 1.** Visualization of mouse retinal vasculature in P30 *Atf6^+/+^* and P30 *Atf6^-/-^* retinas. Retinal wholemounts from P30 were stained for endothelial cells with isolectin B4-Alexa 488. (a) The distribution of vascular plexus between *Atf6^+/+^* and *Atf6^-/-^* wholemount retinas showed no detectable changes. Scale bar 1mm. (b) Illustration of superficial, intermediate, and deep plexuses in the retina. (c) The vascular plexus between *Atf6^+/+^* and *Atf6^-/-^* retinas showed no detectable changes in the superficial, intermediate, and deep layers at this age. Scale bar 50 um.


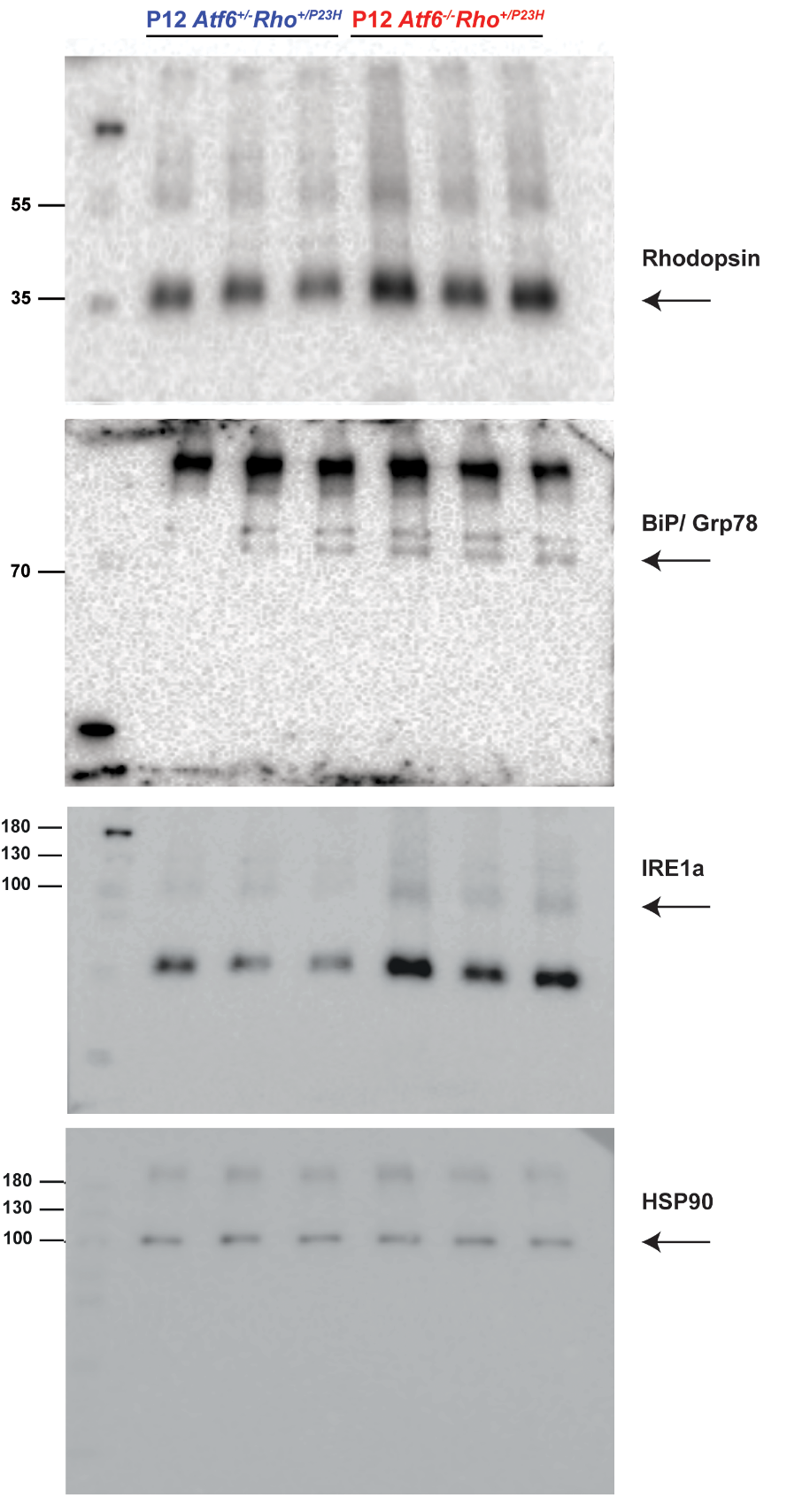


**Supplemental Figure 2**. The entire immunoblot image identifies Rhodopsin-, BiP/Grp78-, IRE1a-, and HSP90-immunoreactive bands in P12 (Figure 1a).


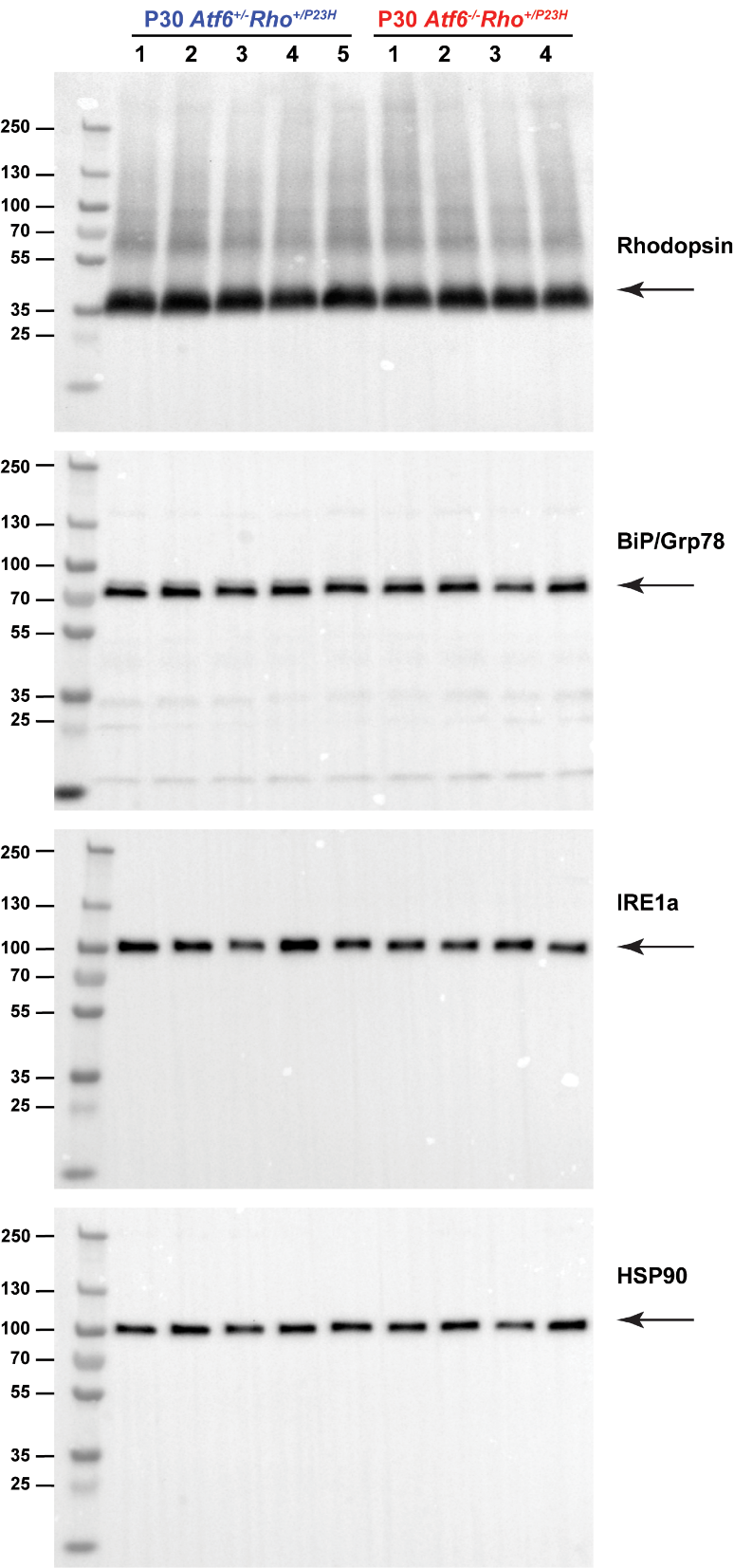


**Supplemental Figure 3**. The entire immunoblot image identifies Rhodopsin-, BiP/Grp78-, IRE1a-, and HSP90-immunoreactive bands in P30 (Figure 3c).


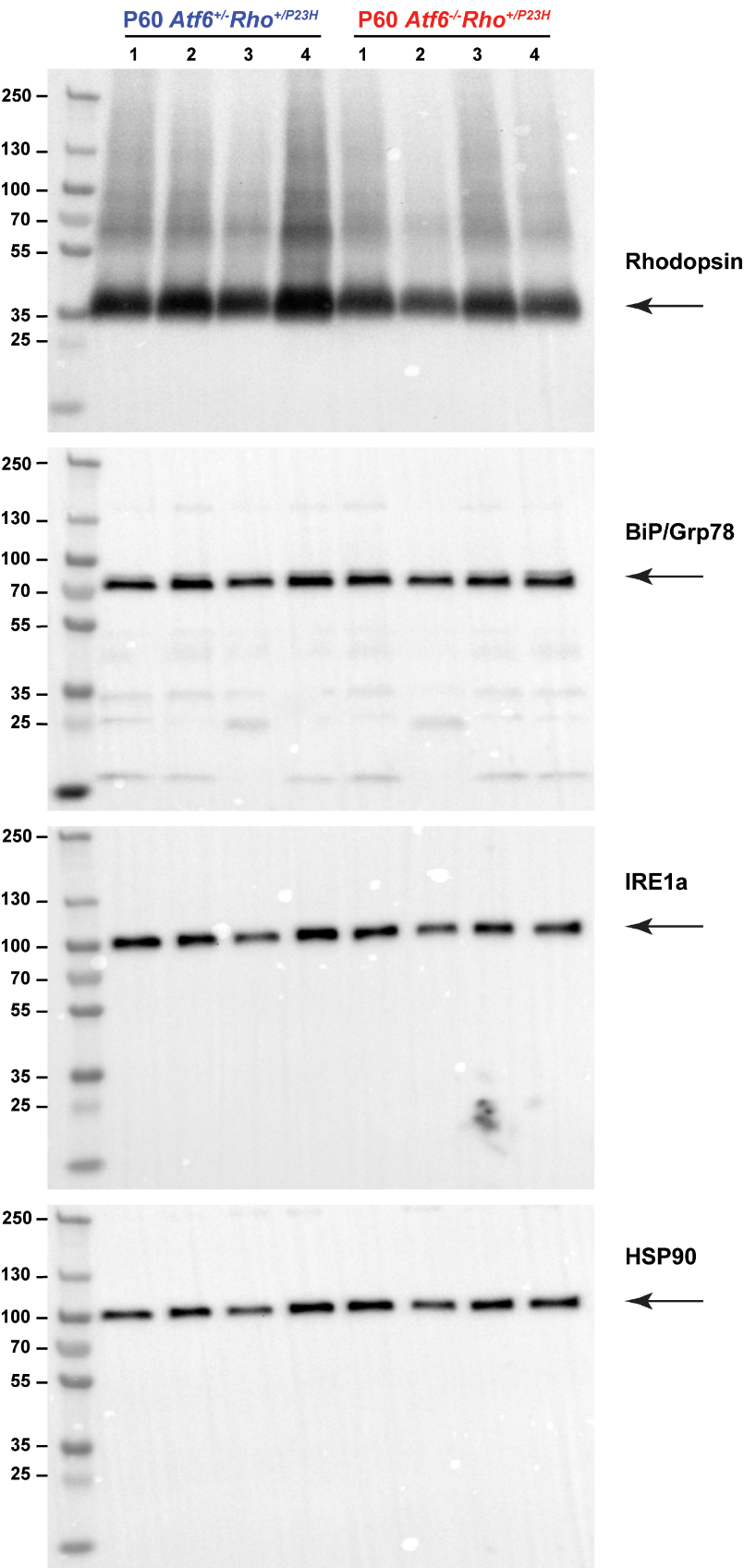


**Supplemental Figure 4**. The entire immunoblot image identifies Rhodopsin-, BiP/Grp78-, IRE1a-, and HSP90-immunoreactive bands in P60 (Figure 4c).
